# Epidemiology of viral respiratory infections in a pediatric reference hospital in Central Panama

**DOI:** 10.1186/s12879-020-05720-1

**Published:** 2021-01-09

**Authors:** Virginia Núñez-Samudio, Iván Landires

**Affiliations:** 1 Instituto de Ciencias Médicas, PO Box 0710-00043, Las Tablas, Los Santos Panama; 2 Sección de Epidemiología, Departamento de Salud Pública, Región de Salud de Herrera, Ministry of Health, Chitré, Panama; 3 Centro Regional Universitario de Azuero (CRUA), Universidad de Panamá, Chitré, Herrera Panama; 4 Hospital Joaquín Pablo Franco Sayas, Región de Salud de Los Santos, Ministry of Health, Las Tablas, Panama

**Keywords:** Acute respiratory infections, Central America, Epidemiology, Panama, Pediatrics, Surveillance, Viruses

## Abstract

**Background:**

Acute respiratory infections (ARIs) are a worldwide public health problem. It is estimated that up to 80% of cases of ARIs are caused by viruses. In Central America, however, we identified few epidemiologic studies on the main ARI-related viruses in hospitalized children.

**Methods:**

This study retrospectively analyzed the clinical charts of patients ages 29 days to 14 years admitted with diagnoses of ARIs in a pediatric reference hospital in central Panama during 2016. The variables analyzed were age, sex, signs, symptoms, and diagnosis at admission. Samples of patients to whom a viral panel was indicated were analyzed via quantitative polymerase chain reaction, qPCR.

**Results:**

The most common virus was respiratory syncytial virus (RSV; 25.9%), followed by influenza A virus (10.6%), rhinovirus (10.6%), parainfluenza type 3 (PIV-3; 8.2%) and adenovirus (5.9%). However, virus detection varied with patient age and season. RSV and Influenza virus were respectively identified mainly during July–November and May–July. All cases of viral co-infection occurred in children < 5-years-old. Both influenza A (H1N1) pdm09 and rhinovirus were detected in all pediatric ages analyzed in this study, unlike RSV and PIV-3, which were only present in children < 5-years-old.

**Conclusions:**

This study analyzed the epidemiological patterns of different respiratory viruses in pediatric patients with ARI from central Panama and found that the prevalence of the specific respiratory viruses identified varied with season and age. The most common viruses were RSV, influenza A, and rhinovirus. There were no reports of human metapneumovirus associated with ARI, which may be explained by the time and geographic location of the study. Knowledge of the local epidemiology of respiratory viruses in tropical countries is helpful in forecasting the peaks of hospitalizations due to ARIs and may help improve prevention efforts aiming at respiratory disease control in these settings.

## Background

Acute respiratory infections (ARIs) constitute a complex group of infections affecting any anatomic site within the respiratory tract. Worldwide, these are a large public health problem, ranking among the top causes of pediatric outpatient and inpatient care [2]. Lower ARIs are considered as one of the top causes of mortality in children < 5-years-old [2–5]. Even though ARIs can be caused by diverse etiologic agents, it is estimated that up to 80% of cases are caused by viruses [6, 7]. Globally, the human respiratory syncytial virus (RSV; family *Pneumoviridae*) is one of the main viruses causing lower ARIs and approximately 45% of the hospitalizations and deaths in children over 6 months old can be attributed to this virus [3]. Other viruses associated with ARIs are rhinovirus (family *Picornaviridae*); parainfluenza 1–4 viruses (PIV; family *Paramyxoviridae*); influenza A, B, and C (family *Orthomyxoviridae*), adenovirus (family *Adenoviridae*), and human metapneumovirus (HMPV; family *Paramyxoviridae*) [8–10].

In Central America, we identified few epidemiologic studies on the main ARI-related viruses in hospitalized children, usually due to diagnostic limitations [11]. Given the availability of vaccines and other preventative measures, the virologic characteristics, and the specific epidemiological behavior in each geographic region, causes of ARIs are dynamic, with some pathogens emerging and others in potential decline. It is noteworthy that among all the vaccines that may prevent pediatric ARIs, Panama’s Expanded Immunization Program (EIP) includes conjugated pneumococcal vaccines at ages 2 and 4 months with a booster at 12 months and influenza vaccines starting at age 6 month, at a 4-week interval and then each year until 5 years of age [12]. According to a Pan-American Health Organization’s 2019 Report on Immunization in the Americas, Panama had an overall vaccine coverage of 95% during the first year of life [13].

In Panama, epidemiologic surveillance for influenza and other respiratory viruses is conducted as part of the National Epidemiologic Surveillance System [14]. Thus, with the aim of having a better understanding of the dynamics of ARI-related viruses in hospitalized children in tropical countries, we conducted a retrospective study analyzing the prevalence and characteristics of respiratory viruses detected in hospitalized children with a diagnosis of ARI in the Pediatric ward of a public reference hospital in central Panama.

## Methods

A retrospective epidemiologic study of the prevalence and characteristics of respiratory viruses in patients admitted between January and December 2016 with a diagnosis of ARI was carried out in the Pediatrics ward of the Cecilio Castillero General Hospital (CCGH) which admits patients aged between 29 days and 14 years. CCGH, located in the city of Chitré, province of Herrera, is the main public pediatric reference center among the four central provinces of the Republic of Panama (namely, Herrera, Los Santos, Coclé, and Veraguas).

Demographic and clinical data of the children hospitalized with a diagnosis of ARI during 2016 were abstracted from the Medical Records Department. The variables analyzed were age, sex, signs, symptoms, and the following admission diagnoses from the *International Statistical Classification of Diseases and Related Health Problems*, 10th Revision (ICD-10) [1]: suspected influenza due to unidentified influenza virus with other respiratory manifestations (J11.1), pneumonia, unspecified organism (J18), acute bronchiolitis (J21) and unspecified asthma with acute exacerbation (J45.901). In case the attending physician ordered a respiratory virus panel, the Hospital Epidemiologic Surveillance Database, a component part of the National Epidemiologic Surveillance System, was also searched for cases matching the name and unique identifier of these patients. The respiratory viruses identified were then included as a study outcome.

To confirm a suspected case of viral ARI at the CCGH under the National Epidemiologic Surveillance System, a nasopharyngeal swab is collected, preserved, and transported to the Central Reference Public Health Laboratory at the Gorgas Memorial Institute for Health Studies in Panama City, Panama. Samples of patients to whom a viral panel was indicated were analyzed via quantitative polymerase chain reaction, qPCR, for the respiratory viruses under national surveillance (influenza A and B, RSV, HMPV, human rhinovirus, adenovirus, and PIV 1, 2, and 3) [14, 15].

Patient’s data were grouped by age in three categories (< 1, 1–4, and 5–14 years). For analyses by sex, we compared absolute number of all age groups of females and males hospitalized with a diagnosis of ARI during the study. Data were captured using MS Excel (Microsoft Corporation; Redmond, WA) spreadsheet and data analyses were conducted using Stata v. 11.0 (StataCorp LLC; College Station, TX). We used Fisher’s exact test to compare proportions (α = 0.05). The prevalence (*P*) of viral ARI among patients admitted was calculated using the formula *P=C*_*v*_*/N*, where *C*_*v*_ is the number of ARI cases with positive viral panel tests for the specific virus *v* and *N* is the number of ARI cases with viral panel samples collected during the study period [16].

The study protocol was reviewed and approved by the Bioethics Committee at the University of Panama (No. CBIUP/328/17).

## Results

During 2016, a total of 217 children were admitted with the diagnosis of ARI to the Pediatrics ward of the CCGH (Table 1). Among the 217 children admitted with the diagnosis of ARI, 109 (50.2%) were male and 108 (49.8%) were female (*P*=0.93) and 30% lived in rural areas while 70% lived in urban areas. One hundred and sixty-seven (77%; 95% CI: 71–80%) cases were admitted between June and August; and 190 (87%; 95% CI: 83–91%) were < 5-years-old. Nineteen (8.7%) patients presented with comorbidities, the most important of which was asthma (12/19). Other comorbidities included sickle-cell anemia (3/19), cerebral palsy (2/19), prematurity (1/19), and laringotracheomalacia (1/19).

**Table 1:** Relationship between age, sex, and clinical presentation of inpatients with ARI and the proportion of patients with a positive viral panel

Patient characteristics	All hospitalized patients with ARI (***N***=217)	Subgroup of patients tested with qPCR viral panel (***N***=85)
**Age group**		
< 1 year	87 (40·1%)	27 (31·8%)
1–4 years	103 (47·5%)	41 (48·2%)
5–14 years	27 (12·4%)	17 (20·0%)
**Sex**		
Male	109 (50·2%)	38(44·7%)
Female	108 (49·8%)	47 (55·3%)
**Signs and Symptoms**		
Fever	150 (69·1%)	67 (78·8%)
Shortness of breath	149 (68·7%)	50 (58·8%)
Dry cough	123 (56·7%)	49(57·6%)
Rhinorrhea	92 (42·4%)	37(43·5%)
Productive cough	43 (19·8%)	15 (17·6%)
Vomiting	34 (15·7%)	13(15·3%)
Diarrhea	19 (8·7%)	9(11·1%)
**Diagnosis at Hospitalization (ICD-10 Code)**		
Suspected influenza due to unidentified influenza virus with other respiratory manifestations (J11.1)	104 (47.9%)	76 (89.4%)
Pneumonia, unspecified organism (J18)	48 (22.1%)	2 (2.4%)
Acute bronchiolitis (J21)	35 (16.1%)	5 (5.9%)
Unspecified asthma with acute exacerbation (J45.901)	30 (13.8%)	2 (2.4%)

*Abbreviations*: *ARI* acute respiratory infection, *ICD-10* International Statistical Classification of Diseases and Related Health Problems, 10th Revision, *influenza A* influenza A (H1N1) pdm09 virus, *PIV 3* parainfluenza type 3 virus, *RSV* respiratory syncytial virus, *qPCR* quantitative polymerase chain reaction

The largest number of patients hospitalized for ARIs presented with fever (69.1%), shortness of breath (68.7%) and dry cough (56.7%) as the most frequent symptoms. The main cause of hospitalization was suspected influenza due to unidentified influenza virus with other respiratory manifestations (Table 1). Among all patients under 5 years of age, half (49.5%) had received an influenza vaccine that year.

**Fig. 1:**
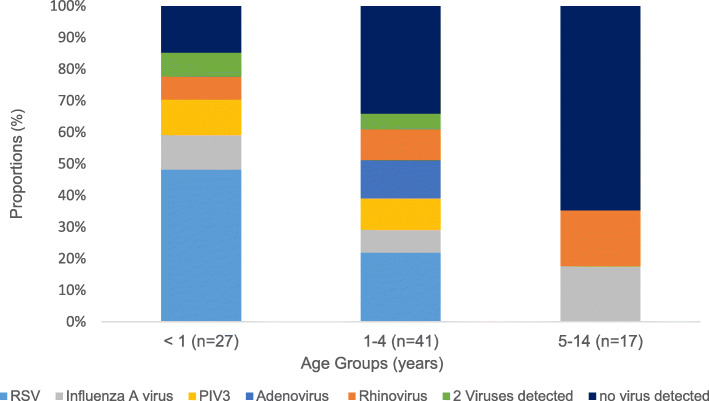
Frequency of viral pathogens detected on nasopharyngeal/oropharyngeal swab by age group. The main virus detected in the samples analyzed for the groups of < 1 year and 1–4 years was the RSV, detected in 48 and 22% respectively. In the group of 5–14 years of age, Influenza A and Rhinovirus were detected, accounting for 35% of the samples analyzed. Abbreviations: Influenza A virus,: influenza A (H1N1) pdm09; PIV, parainfluenza type 3 virus; RSV, respiratory syncytial virus

Among all patients admitted with a diagnosis of ARI, 85 (39%; 95% CI: 33–46) had the epidemiologic surveillance qPCR respiratory virus laboratory panel. Among them, 47 (55%; 95% CI: 44–66) were males and 38 (45%; 95% CI: 34–55) females. Seventy-six (89%; 95% CI: 83–96) of the patients tested with qPCR viral panel were admitted with the diagnosis of suspected influenza due to unidentified influenza virus with other respiratory manifestations while nine were admitted with the diagnoses of acute bronchiolitis (*n*=5), pneumonia (*n*=2), and asthma crisis (*n*=2). The 85 samples analyzed were from patients across all age groups: < 1 year (*n*=27), 1–4 years (*n*=41), and 5–14 years (*n*=17). Positive results were obtained for the viruses under epidemiologic surveillance in 56 (66%; 95% CI: 55–76%) of the 85 cases, while 29 (34%) samples were negative. The test positivity proportions for viruses identified across all age groups were 25.9% for RSV, 10.6% for influenza A (H1N1) pdm09, 10.6% for rhinovirus, 8.2% for PIV-3, and 5.9% for adenovirus. No infections by HMPV were detected. The test positivity proportions for viral co-infections was 4.7% and it was confirmed in four samples analyzed for children < 5-years. All four co-infections included the PIV-3; three were co-infections with RSV and one with adenovirus. In the group of patients < 1-year-old there was an 85.2% positivity proportion, while this number was 65.6% among the 1- to 4-year-olds group and 35.3% among the 5- to 14-year-olds. The variation of positivity proportions of viruses detected among the three age groups was statistically significant (*P*=0.003). The viruses most frequently detected in the group of patients < 1-year-old were RSV (*n*=13), influenza A (*n*=3), PIV 3 (*n*=3), rhinovirus (*n*=2), and co-infection with two viruses (*n*=2). In the group of patients 1–4 years of age, we observed RSV (*n*=9), PIV 3 (*n*=4), adenovirus (*n*=5), rhinovirus (*n*=4), influenza A (*n*=3), and co-infection with two viruses (*n*=2). The 5–14–years group had only tested positive for influenza A and rhinovirus (*n*=3 each) (Fig. 1). Comparison of the RSV proportions between the three age groups showed a statistically significantly difference (*P*=0.02).

**Fig. 2:**
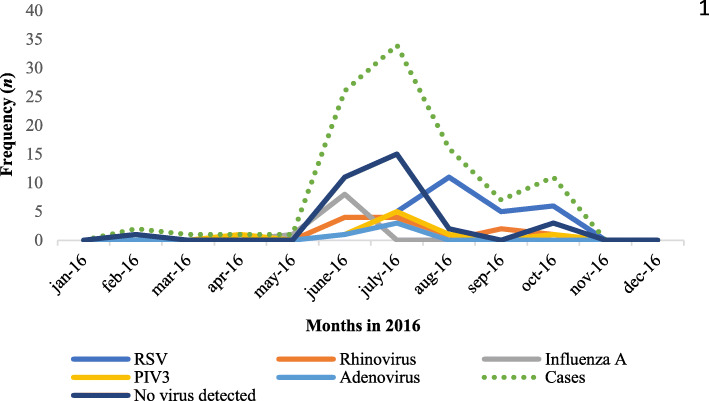
Detection of respiratory viruses by month of the year 2016. This figure shows that from May to November is the period where the majority of hospitalizations for ARIs occur. Influenza A peaked from May to July while RSV peaked from July to November. The ARIs for adenovirus, PIV-3, and rhinovirus were also increasing in smaller proportion from May to November. Abbreviations: ARIs: Acute respiratory infections, Influenza A virus, influenza A (H1N1) pdm09; PIV, parainfluenza type 3 virus; RSV, respiratory syncytial virus. Case, total number of cases with acute respiratory infection where a viral panel was assessed

Regarding the seasonal detection of respiratory viruses, influenza A (H1N1) pdm09 peaked in June, while RSV peaked in August (Fig. 2). Both influenza A (H1N1) pdm09 and rhinovirus were detected in all pediatric ages analyzed in this study, unlike RSV and PIV-3, which were only present in children < 5-years-old.

## Discussion

This study describes the epidemiology of respiratory viruses in children admitted with the diagnosis of ARI in a reference hospital in central Panama. ARIs, at 45% of total admissions, constituted one of the top diagnoses among hospitalized children < 14-years old—especially among children < 5-years-old—without overall statistically significant differences by sex [3]. Earlier studies with inpatients in the Central American region showed that respiratory viruses are responsible for 52.6% of all the ARI cases, affecting mostly children < 5-years-old [11].

A virus was identified in about half (54.1%) of the samples analyzed. Studies conducted in hospitalized patients have evidenced positivity rates between 36 and 85% [6, 17]. We observed that the proportions of the specific respiratory viruses identified varied by age and month of the year (Figs. 1 and 2, respectively). The overall prevalence of RSV was 25.9%, but it was higher (48%) for children < 1-year-old. These findings resemble those from a study in Guatemala, where the prevalence of RSV in hospitalized children was 26.4%, and it was higher (71.8%) for children < 1-year-old [11]. The relatively higher prevalence of RSV as a causative agent of ARI in hospitalized children < 5-years-old has also been documented [5].

Influenza A (H1N1) pdm09 and rhinovirus each had a prevalence of 10.6%. Both viruses were detected in the three age groups, unlike RSV and PIV-3, which were only present in children < 5-years-old. Previous studies in low- and middle-income countries have also described these viruses as relatively common among patients hospitalized with ARI [17, 18].

The prevalence of viral co-infection was 5%. All the co-infections occurred in children < 5-years-old and all of them occurred with PIV-3. Previous studies detected co-infections at similar rates, mostly due to two viruses (82.1%), with a high percentage of positive results in younger children, probably due to low viral elimination by the developing immune system [8, 11]. The proportion of children with co-infections in this study is similar to other studies in Central America [19].

The most frequent viruses identified were RSV (25.9%), influenza A (10.6%), and rhinovirus (10.6%). We identified RSV from July–November, with the peak in August, while influenza virus was identified in May–July, with the peak in June. This variation is in accordance with that reported from other tropical countries [16]. Panama has a dry, tropical climate [20]. In Central America, the annual precipitation cycle can be approximately divided in a dry season from December to April and in a rainy season from May to November [21]. Increased precipitation and humidity might be factors in the transmission of respiratory viruses [22] and it has been hypothesized that tropical rainy seasons may encourage the transmissibility of RSV more than that of influenza [21].

Although, HMPV was not reported in this study, it is a leading cause of ARIs in other studies from Central America [19, 23]. Metapneumovirus is interesting, as the virus is associated with high morbidity in mainly high-income countries [24, 25] but also was recently found in high numbers in the PERCH multi-center study of pneumonia in low-income countries [26]. Panama included HMPV surveillance in 2010 as part of the National Epidemiologic Surveillance System. A previous study [27] described a HMPV frequency among ≤5-year-olds hospitalized patients with ARI of 1.3% in 2011 and 13.9% in 2012, which may suggest important fluctuations over time. We did not detect HMPV in hospitalized children with ARI in 2016. A possible explanation is variability over time and geographic location of respiratory pathogens.

Vaccination against influenza was included in Panama’s Broadened Immunization Program from 2010 and it is administered every year starting in April to children between 6 and 35 months of age and any person, regardless of their age, who presents with a chronic health condition [28]. We have found that at the time of their admission, an important number of children were vaccinated against influenza (49.5%), which could have contributed to the lower frequency of cases of ARI due to influenza A (H1N1) pdm09.

This study used the WHO criteria to classify ARIs [29] and included patients under 5 years of age hospitalized with ARI. Thus, an important limitation is the limited generalizability to children with ARI who do not require hospitalization. According to the WHO classification, although the group of patients older than 5 years was hospitalized, their condition could not be classified as severe ARI. This limitation aside, this study provides important information in the epidemilogic behavior of ARIs in a central american tropical country.

## Conclusions

This study analyzed the epidemiological patterns of different respiratory viruses in pediatric patients with ARI from central Panama and found that the prevalence of the specific respiratory viruses varied with season and age. In addition RSV, influenza A virus and rhinovirus are the most common viruses. Knowledge of the local epidemiology of tropical climate regions is helpful in forecasting the peaks of respiratory viruses associated with hospitalization due to ARI. This in turn could translate into improved prevention efforts aiming at respiratory disease control, including hospital measures to improve infection control [30].

## Data Availability

The datasets used and analyzed are available from the corresponding author on reasonable request.

## Abbreviations

ARIs: Acute respiratory infections
CCGH: Cecilio Castillero General Hospital
EIP: Expanded Immunizatio Program
ICD-10: *International Statistical Classification of Diseases and Related Health Problems*, 10th Revision [1]
PIV: Parainfluenza virus
RSV: Respiratory syncytial virus
qPCR: quantitative polymerase chain reaction
